# The conjugating green alga *Zygnema* sp. (Zygnematophyceae) from the Arctic shows high frost tolerance in mature cells (pre-akinetes)

**DOI:** 10.1007/s00709-019-01404-z

**Published:** 2019-07-10

**Authors:** Kateřina Trumhová, Andreas Holzinger, Sabrina Obwegeser, Gilbert Neuner, Martina Pichrtová

**Affiliations:** 1 Faculty of Science, Department of Botany, Charles University, Benátská 2, 128 00 Prague, Czech Republic; 2 Institute of Botany, Functional Plant Biology, University of Innsbruck, Sternwartestraße 15, 6020 Innsbruck, Austria

**Keywords:** Auramine O, Chlorophyll fluorescence, Freezing, Ice, Live cell staining, Ultrastructure

## Abstract

Green algae of the genus *Zygnema* form extensive mats and produce large amounts of biomass in shallow freshwater habitats. Environmental stresses including freezing may perturb these mats, which usually have only annual character. To estimate the limits of survival at subzero temperatures, freezing resistance of young *Zygnema* sp. (strain MP2011Skan) cells and pre-akinetes was investigated. Young, 2-week-old cultures were exposed to temperatures of 0 to – 14 °C at 2-K steps, whereas 8-month-old cultures were frozen from − 10 to – 70 °C at 10-K intervals. Cell viability after freezing was determined by 0.1% auramine O vital fluorescence staining and measurements of the effective quantum yield of photosystem II (*Ф*_PSII_). At – 8 °C, the young vegetative cells were unable to recover from severe frost damage. But temperatures even slightly below zero (− 2 °C) negatively affected the cells’ physiology. Single pre-akinetes could survive even at – 70 °C, but their LT_50_ value was − 26.2 °C. Severe freezing cytorrhysis was observed via cryo-microscopy at – 10 °C, a temperature found to be lethal for young cells. The ultrastructure of young cells appeared unchanged at – 2 °C, but severe damage to biomembranes and formation of small foamy vacuoles was observed at – 10 °C. Pre-akinetes did not show ultrastructural changes at – 20 °C; however, vacuolization increased, and gas bubbles appeared at – 70 °C. Our results demonstrate that the formation of pre-akinetes increases freezing resistance. This adaptation is crucial for surviving the harsh temperature conditions prevailing in the High Arctic in winter and a key feature in seasonal dynamics of *Zygnema* sp.

**Electronic supplementary material:**

The online version of this article (10.1007/s00709-019-01404-z) contains supplementary material, which is available to authorized users.

## Introduction

Polar hydro-terrestrial ecosystems are characterized by changing and harsh environmental conditions. Freezing and desiccation are the primary environmental stresses in such habitats, although osmotic stress, high UV irradiation, nutrient deficiency, and seasonal changes in irradiance can also negatively affect the organisms living there. Algae and cyanobacteria are the main primary producers in polar ecosystems. They have to cope with long winters and even during growth periods, they may be exposed to frequent freeze-thaw cycles (Davey 1989; Thomas et al. 2008). During freezing and thawing, their cells are exposed to a radically changing environment, which leads to cryoinjuries (damages caused by frost). Depending on the cooling rate, two different damaging mechanisms can occur: freezing cytorrhysis (collapse of a cell wall due to the loss of water through osmosis) caused by considerable cell dehydration or mechanical disruption of membranes (Acker and McGann 2003).

Terrestrial algae inhabiting this harsh environment had to adapt to these stresses. One of the most widespread adaptation strategies is represented by specialized, stress-resistant life cycle stages, for example, oospores of *Chara* sp. (Proctor 1967), aplanospores of *Coleochaete* (Delwiche et al. 1989), and various specialized life cycle stages of green algae (reviewed in Leliaert et al. 2012), whereby reduced rates of physiological activity are complemented by additional protective mechanisms, i.e., changes in membrane composition or production of special protective substances (summarized in Elster 1999, Holzinger and Pichrtová 2016). Nevertheless, many algae survive stress in a vegetative state, for example, *Micrasterias denticulata* Brébisson ex Ralfs (Meindl et al. 1989) or tundra stream algae (Sheath et al. 1996).

*Zygnema* is a common alga in polar hydro-terrestrial environments. As other filamentous representatives of the order Zygnematales (Zygnematophyceae), it forms extensive mats during the spring and summer seasons. Such mats were reported and studied mainly in localities fed by slow meltwater streams in the High Arctic (Holzinger et al. 2009, 2011; Pichrtová et al. 2014a, 2016b, 2018) and Antarctic regions (Hawes 1989, 1990). In the life cycle of *Zygnema,* different cell types can be distinguished. Highly resistant zygospores are formed by a sexual process called conjugation, although they are very rarely observed in the polar regions (Elster et al. 1997; Pichrtová et al. 2018). Asexual resistant stages known from the genus (parthenospores, akinetes, and aplanospores; Stancheva et al. 2012) have not been reported from the polar regions yet. However, young vegetative cells develop during the summer season into hardened mature cells termed pre-akinetes; these are characterized by smaller vacuoles, thicker cell walls, increased lipids, and also their typical stellate chloroplasts are reduced (McLean and Pessoney 1970; Pichrtová et al. 2014a, b; Pichrtová et al. 2016a, b). The transition between young vegetative cells and pre-akinetes and *vice versa* is a continuous process (Herburger et al. 2015).

Numerous studies have focused on the ecophysiology and stress resistance of *Zygnema*. Pre-akinetes have been found to play a key role in the survival of these algae, as they are resistant to desiccation (Pickett-Heaps 1975; Pichrtová et al. 2014b; Herburger et al. 2015; Rippin et al. 2017), osmotic stress (Kaplan et al. 2013; Pichrtová et al. 2014a), and UV radiation; however, younger cells have a more flexible reaction to UV (Holzinger et al. 2009; Holzinger et al. 2018). Moreover, *Zygnema* is a representative genus commonly studied for its adaptation to terrestrial life because Zygnematophyceaean algae are considered to be the closest relatives of land plants (Wodniok et al. 2011; Zhong et al. 2014; de Vries et al. 2016; Lemieux et al. 2016; de Vries et al. 2018). Surprisingly, little is known about the role of pre-akinete formation in the overwintering strategy and freezing resistance of *Zygnema*. Living pre-akinetes embedded in ice were observed during the Arctic winter (Pichrtová et al. 2016b). Although these cells were capable of restoring their photosynthetic activity right after thawing, a large amount of dead algal biomass was found at the study site after winter (Pichrtová et al. 2016b). Hawes (1990) investigated the freezing tolerance of both natural samples from Antarctica and cultures. He reported that all cells from the field resembled pre-akinetes. Repeated freeze-thaw cycles had no effect on the photosynthetic rate. In addition, a small proportion of cells tolerated long-term (120 days) exposure to – 20 °C, suggesting their ability to survive long-term exposure to sub-zero temperatures during the Antarctic winter. However, no mechanism underlying this phenomenon has been proposed (Hawes 1990).

The present study focused on freezing resistance by an Arctic *Zygnema* sp. The formation of pre-akinetes was previously observed in the Arctic at the end of the growing season (Pichrtová et al. 2014a) and cells could overwinter in this state (Pichrtová et al. 2016b). Based on field observations, we aimed to estimate the limits of survival of young cells and pre-akinetes. Three experiments with cultures of different ages were performed. Pre-akinetes were hypothesized to resist much lower freezing temperatures than young vegetative cells. Additionally, young cells were exposed to a freeze-thaw cycle to test if repeated freezing could harm them more. Changes in photosynthetic activity before and after freezing experiments were measured by the effective quantum yield of photosystem II (*Φ*_PSII_). Frost injuries were observed via light and transmission electron microscopy. Moreover, the freezing process of young vegetative cells was investigated by cryo-microscopy to evaluate changes in morphology during the formation of ice. The results are discussed in the context of stress resistance and the seasonal dynamics of this ecologically important alga.

## Methods

### Algal material

Experiments were conducted with the Arctic *Zygnema* sp. strain MP2011Skan (Pichrtová et al. 2018: Table 1 Locality 4, Skansbukta, culture isolated from a field sample collected on August 19, 2011) whose *rbc*L sequence is identical to that of *Zygnema* sp. B (CCALA 976) and GenBank accession numbers JX075101 (Kaplan et al. 2013) and LN611664 (Pichrtová et al. 2014b). Cultures were maintained in Bold’s Basal Medium (BBM; Bischoff and Bold, 1963) solidified with 1.5% agar and incubated at a light/dark regime of 16/8 h at 20 °C and ~ 33 μmol photons m^−2^ s^−1^. Cultures at two different stages were used for the experiments: (1) young vegetative cells obtained 2 weeks after inoculation and (2) eight-months-old pre-akinetes. The occurrence of pre-akinetes and the general condition of cultures was examined microscopically (Axiovert 200 M, Carl Zeiss AG, Jena, Germany) before the experiments.

**Table 1:** The values of *Φ*_PSII_ in 1h during recovery period were compared with values before freezing treatment using paired t-tests

Young cells—single freezing			
*N*, 24			
Before freezing		24 h after freezing	
Mean	0.60562	Mean	0.19687
Median	0.602	Median	0.125
*t*			9.036
*p* (same)			0.000000004995
Young cells—double freezing			
*N*, 9			
Before freezing		24 h after freezing	
Mean	0.56044	Mean	0.29633
Median	0.561	Median	0.392
*t*			4.181
*p* (same)			0.003077
Pre-akinetes			
*N*, 21			
Before freezing		24 h after freezing	
Mean	0.38195	Mean	0.089667
Median	0.352	Median	0
*t*			8.012
*p* (same)			0.000000114

Values were compared using paired *t* tests. Each of the three experiments was tested separetly.

### Experimental freezing

Freezing experiments were carried out in commercial chest freezers (GT 2102, Liebherr, Lienz, Austria) during spring 2017. The temperature inside the freezing compartment was controlled as described by Kuprian et al. (2016). Three different cooling experiments were performed. In the first experiment (1), young cultures were exposed to eight experimental temperatures from 0 °C to – 14 °C for 10 h, with a cooling and warming rate of 4 K h^-1^. In the second experiment (2), the cells were frozen in two consecutive freezing cycles, both with exposure time of 8 h. Three experimental temperatures were investigated: − 4°C, − 6°C, and − 8°C with cooling and warming rates of 4 Kh^-1^. Between cycles, samples were kept in the cultivation chamber at + 10 °C and ~ 33 μmol photons m^−2^ s^−1^. The third experiment (3) was performed with mature cells (pre-akinetes) at seven experimental temperatures from – 10 °C to – 70 °C, exposure time of 8 h, and cooling and warming rate of 4 K h^-1^. Controls without experimental treatment were kept in the cultivation chamber as described above. Detailed parameters of freezing cycles are given in Online resource 1.

A comparable amount of fresh biomass (expressed as average ± standard deviation) was used for the experiments: 0.003 ± 0.0008 g for young cells and 0.007 ± 0.0013 g for pre-akinetes (analytic balance, Sartorius, Göttingen, Germany). The experiment was performed in three independent biological replicates for each treatment. Cells were transferred onto glass microfiber filters GF/C Ø 47 mm (Whatman, UK), which were placed on filter paper Ø 90 mm (Whatman, UK) in a plastic Petri dish. The space between the microfiber filter with algal biomass and the wall of the Petri dish was covered with a tablespoon of crushed ice. To prevent artificial supercooling of algal cells, ice nucleation was triggered around − 1.5 °C by applying 400 μL of ice nucleation active bacterial suspension (*Pseudomonas syringae* van Hall) on the ice-covered part of the filter paper. This method is well established for higher plants (e.g., Hacker and Neuner 2007; Kuprian et al. 2016), but has been used here for the first time on algae. The algae were never mixed with the bacterial suspension, but the latter was applied on ice in the vicinity of the algal biomass. Direct application to filaments of *Zygnema* sp. caused cell death within a few hours during the recovery period. Additionally, thermocouples were mounted in the proximity of cells to monitor the temperature during experimental freezing cycles. The filter paper was removed right after freezing, and only the glass microfiber filter with algae was left in the Petri dish sealed with parafilm. Samples were placed under continuous illumination (~ 50 μmol photons m^-2^ s^-1^) at around + 19 °C and rewetted with 200 μL water 6 h and 22 h after treatment to prevent additional desiccation stress.

Small aliquots of cultures containing pre-akinetes were frozen to – 50 °C and – 70 °C and, after the experiments, were transferred to Petri dishes with BBM media (Bischoff and Bold 1963) solidified with agar, and incubated at a light/dark regime of 16/8 h at + 20 °C and ~ 33 μmol photons m^−2^ s^−1^ for 3 weeks. These cells were microscopically examined (Zeiss Axiovert 200 M) for the presence of living cells after 2 and 4 weeks of cultivation.

### Measurement of the effective quantum yield

The effective quantum yield of photochemical energy conversion in PSII (*Φ*_PSII_) was measured using a PAM 2500 fluorometer (Heinz Walz GmbH, Effeltrich, Germany). *Φ*_PSII_ is a relative parameter computed as (F_M_’ – F)/F_M_,’ where F is steady-state fluorescence in the light-adapted state and F_M_’ the maximum fluorescence in the light-adapted state measured after the application of a saturation pulse. The first measurement was taken before experiments, after placing the filters with the algal material into plastic Petri dishes. Subsequent measurements were performed 1 h, 3 h, 6 h, and 24 h after the end of freezing experiments.

### Vital staining and light microscopy

Algal samples were examined by light or fluorescence microscopy (Zeiss Axiovert 200 M) using a Zeiss Axiocam HRc camera and a Zeiss filter set 09 (for fluorescence microscopy). A small amount of biomass was stained with 0.1% auramine O (Sigma Aldrich, Steinheim, Germany) to estimate the viability of the cells directly after thawing. Auramine O stains the endomembrane system of metabolically active cells in bright yellow-greenish color (Harris and Gates 1984; Hawes and Davey 1989; Hawes 1990). Staining was performed in 1.5-mL Eppendorf tubes in 1 mL of staining solution for 10 min in darkness. At least twenty images were taken with approximately 600 cells per experimental temperature. The fluorescent cells were counted in ImageJ with the Cell counter plugin (Rasband 2016). At least 500 active/non-active cells were counted for every experimental temperature and control.

### Transmission electron microscopy

Transmission electron microscopy was performed with young cells after a single freezing experiment at – 2 °C and – 10 °C and with old cultures (pre-akinetes) after freezing to – 20 °C and – 70 °C, plus the respective untreated controls. Chemical fixation of filaments was as described by Holzinger et al. (2009) with some modifications. Briefly, samples were fixed in 2.5% glutaraldehyde in 25 mM sodium cacodylate buffer (pH 6.8) for 1.5 h, post-fixed with 1% OsO_4_ at 4°C for 12 h, rinsed and dehydrated in increasing ethanol concentrations, transferred *via* propylene oxide, and embedded in modified Spurr’s embedding resin (Science Services, Munich, Germany). Ultrathin sections were prepared with an Ultra-microtome (Leica Microsystems GmbH, Wetzlar, Germany), counterstained with 2% uranyl acetate and Reynold’s lead citrate, and investigated on a Zeiss LIBRA 120 transmission electron microscope (Carl Zeiss AG, Oberkochen, Germany) at 80 kV. Images were taken with a TRS 2 k SSCCD camera and processed with Adobe Photoshop 7.0 software (Adobe Systems Inc., San Jose, CA, USA).

### Cryo-microscopy

Cryo-microscopical observation was performed on a light microscope (DM1000, Leica Microsystems GmbH) equipped with a cooled slide holder and Leica EC3 camera. Cooling was performed by a custom-built cryo-stage filled with ethanol, which was pumped through the slide holder. The temperature on the sample slide was monitored by thermocouples. Cryo-stage and holder were controlled by custom-designed “Cryostage” software developed by Dr. Othmar Buchner (University of Innsbruck, Austria). Only 2-week-old cells were investigated with this system. The cells were frozen to – 10 °C and images were taken at minute-intervals during freezing until the filament was completely embedded in solid ice. Images were acquired with Leica LAS EZ software and further processed in Adobe Photoshop 7.0.

### Freezing resistance

Lethal temperature at 50% frost damage (LT_50_), a measure of freezing resistance, was determined in two ways. In the first, auramine O staining was used to count the number of viable cells. The LT_50_ value indicated the temperature at which 50% of cells did not show any metabolic activity 1 h after the end of the freezing treatment. In the second, LT_50_ was calculated from *Φ*_PSII_. All *Φ*_PSII_ values were expressed as a percentage of the initial value. The resulting value represented the freezing temperature whereby chlorophyll fluorescence was at 50% of the maximum 24 h after the experiment. LT_50_ was calculated using Boltzmann’s function in Origin 2017 software (Origin Lab Corporation, Northampton, MA, USA).

### Statistical analyses

The effect of experimental temperature and time of recovery on *Φ*_PSII_ values was evaluated using two-way ANOVA with repeated measurements in each of the three experiments. Differences between *Φ*_PSII_ values before and 24 h after treatment were tested with a two-sample *t* value test. For all analyses, the significance value was set as *p* ˂ 0.05. All tests were carried out in the PAST statistical program (Hammer et al. 2001).

## Results

### Physiological performance after freezing

**Fig. 1:**
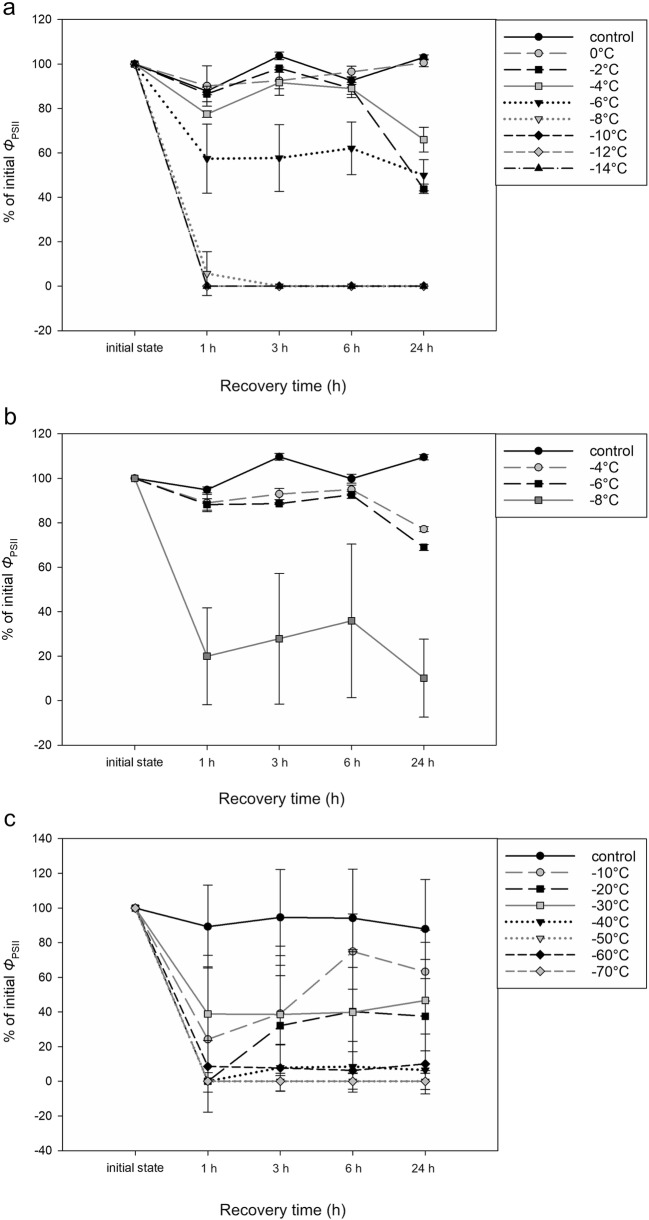
Changes in steady-state quantum yield of PSII in the light (*Ф*_PSII_) of *Zygnema* sp. (strain MP2011Skan) measured before the experiments and during the recovery period. Values relative to the initial values before the experiment are shown (mean ± standard deviation): **a** young cell, single freezing cycle; **b** young cells, double freezing cycle; **c** pre-akinetes

**Fig. 2:**
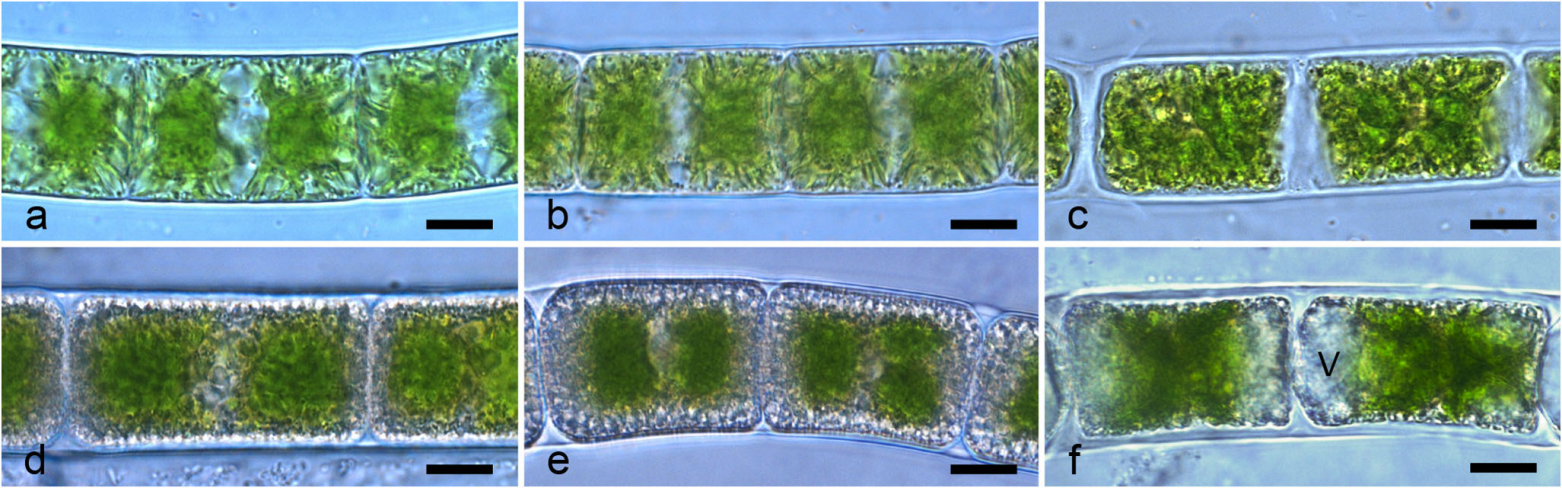
Light micrographs of *Zygnema* sp. (strain MP2011Skan) before and after experimental freezing: **a**–**c**, young cultures (**a** control; **b** – 2 °C; **c** – 10 °C); **d**–**f** pre-akinetes (**d** control; **e** – 20 °C; **f** – 70 °C, *V* vacuole). Scale bars, 10 μm

Mean *Φ*_PSII_ values (± standard deviation) before the experiment were higher in young vegetative cells (0.60 ± 0.02) than in mature pre-akinetes (0.38 ± 0.09). For both types of cells, the initial *Φ*_PSII_ values dropped significantly after experimental freezing (two-sample *t* value test, Table 1). In general, a lower freezing temperature caused a more pronounced decline in *Φ*_PSII_ during the recovery period (Fig. 1). Notably, *Φ*_PSII_ decreased considerably between – 6 °C and – 8 °C in both experiments with young cells (Fig. 1a–b). However, the effect of experimental temperature on *Φ*_PSII_ was statistically significant (*p* < 0.05) only in the single freezing experiment (Table 2). Freezing temperature also significantly affected the *Φ*_PSII_ value of pre-akinetes (Table 2) (Fig. 2).

**Table 2:** Summary of the results of two-way ANOVA with repeated measurements (variable: *Φ*_PSII_ values; factors: recovery time, temperature)

Effect	Sumsqrs	df	Meansqr	*F*	*p*
Young cells—single freezing					
Recovery time	0.05187	3	0.01729	0.007971	0.9989
Temperature	6.813	8	0.8517	6.506	0.0007648
Interaction	0.2327	24	0.009694	− 0.02993	1
Young cells—double freezing					
Recovery time	0.05217	3	0.01739	0.009226	0.9986
Temperature	1.453	3	0.4843	3.71	0.08061
Interaction	0.05039	9	0.005599	− 0.008379	1
Pre-akinetes					
Recovery time	0.03498	3	0.01166	0.03643	0.9898
Temperature	1.261	7	0.1801	11.53	0.00007394
Interaction	0.08617	21	0.004103	− 0.1038	1

Tested *Φ*_PSII_ values are from four dependent measurements during the recovery time (1 h, 3 h, 6 h, and 24 h). Significant interactions between the factors were not encountered.

### Estimation of viability

**Fig. 3:**
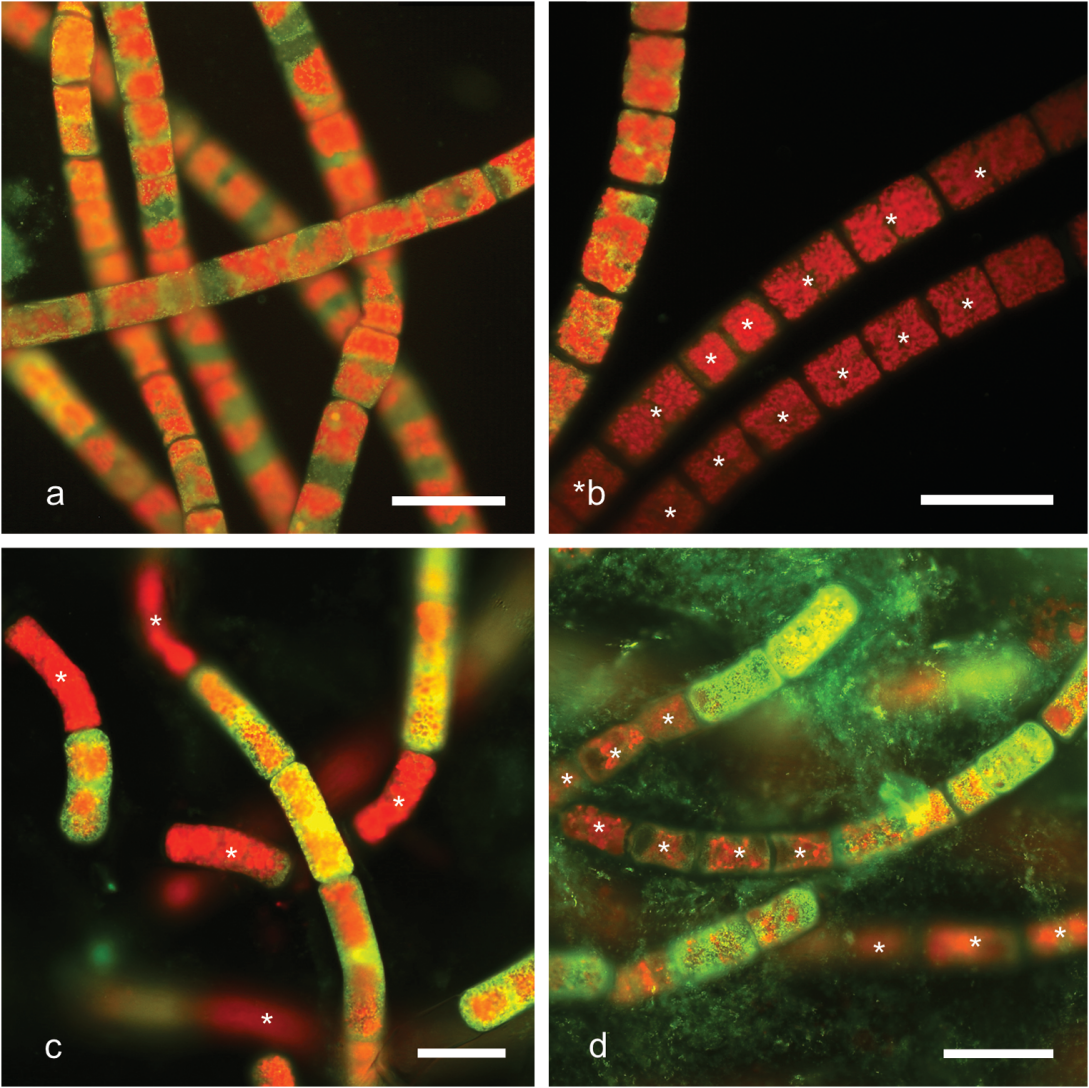
Microphotographs of *Zygnema* sp. (strain MP2011Skan) stained with 0.1% auramine O. Dead cells are marked with asterisks. **a**, **b** young cultures (**a** – 2 °C; **b** – 10 °C); **c**, **d** pre-akinetes (− 20 °C; **d** – 70 °C). Scale bars, 40 μm

Staining with auramine O was used to estimate the number of viable cells after freezing (Fig. 3a–d). Viable cells with a yellow-greenish fluorescence (Fig. 3a) were clearly distinguishable from dead cells exhibiting only red chloroplast autofluorescence (Fig. 3b–d; asterisks). Staining intensity was the same in young cells and pre-akinetes. Moreover, filaments surrounded by mucilage were more metabolically active than separately laying ones. Bacteria in mucilage envelopes were stained green and marked the amount of mucilage surrounding the cells. Pre-akinetes had much more mucilage around them than young vegetative cells (Fig. 3d).

Pre-akinetes frozen to – 70 °C and – 50 °C were additionally transferred to BBM agar medium. Even though there was no detectable *Φ*_PSII_ in the specimens after freezing, 10–20% of cells survived both treatments, as they were able to recover after 4 weeks. However, most of the biomass was dead.

### LT_50_

LT_50_ values calculated from *Φ*_PSII_ were − 5.9 °C for the single freezing experiment and − 6.5 °C for the double freezing experiment (Table 3). No photosynthetic activity could be detected below – 10 °C. Pre-akinetes were more resistant to freezing temperatures than young cells. Their LT_50_ value obtained by *Φ*_PSII_ was determined to be − 26.2 °C.

**Table 3:** LT_50_ values of *Zygnema* sp. (strain MP2011Skan).

	Young cells—single freezing	Young cells—double freezing	Pre-akinetes
LT_50_ values for ФPSII	− 5.9°C	− 6.5°C	− 26.2°C
LT_50_ values for viable cells	− 8.6°C	− 5°C	− 26.1°C

Numbers were determined from Boltzmann’s function fitted to the number of viable cells after experimental freezing and stained with Auramine O or to *Ф*_PSII_ values. Effective quantum yield values were recalculated as a percentage of initial values.

**Fig. 4:**
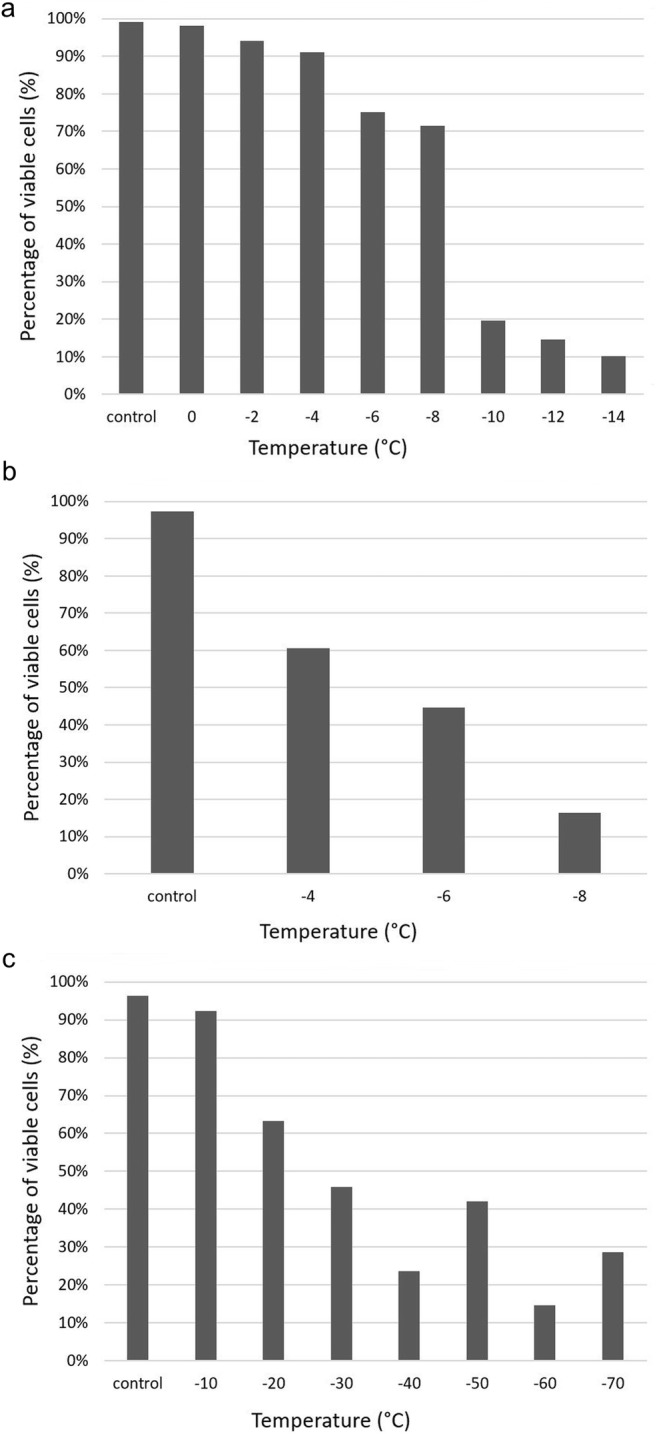
Percentage of viable cells 1 h after freezing experiments. Cells were stained with auramine O to detect viability

Based on the numbers of viable cells stained with auramine O, LT_50_ values were − 8.6 °C for the single freezing experiment and – 5 °C for the double freezing experiment (Table 3). A large difference in the number of viable cells was observed between these two experiments. After freezing to – 8 °C, 71.5% of cells remained active in the single freezing experiment (Fig. 4), but only 16.5% did so after the double freezing experiment. The LT_50_ value was − 26.1 °C for pre-akinetes (Table 3).

### Light microscopy investigations

Young control cells contained two stellate chloroplasts and many small parietal vacuoles. In contrast, pre-akinetes had small chloroplasts, lipid bodies, and a thicker cell wall. The morphology of young cells changed profoundly at experimental temperatures below – 10 °C, but remained unchanged compared to controls at temperatures slightly below 0 °C (Fig. 2a–c). More than 80% of young cells were damaged at – 10 °C; their protoplast was shrunken and detached from the cell wall, and it appeared darker than in untreated controls, sometimes with a yellowish tinge. The number of damaged cells increased as the experimental temperature decreased for both cell types. Pre-akinetes (Fig. 2d–f) exhibited similar damage as young cells, with a shrunken and darker protoplast (Fig. 2f). In pre-akinetes, cells with shrunken protoplasts started to appear in samples frozen to – 20 °C and their number increased further as the temperature became even lower. Still, most cells frozen down to – 20 °C maintained a normal morphology (Fig. 2e). Moreover, a clear tendency to form vacuoles in the protoplasts was observed after freezing cycles at temperatures below – 30 °C. The vacuoles were localized in the apical part of the cells (Fig. 2f). We also observed air bubbles forming inside thawed cells previously frozen to temperatures below – 30 °C. The number of cells with vacuoles and gas bubbles increased further at even lower temperatures.

### Cryo-microscopy

**Fig. 5:**
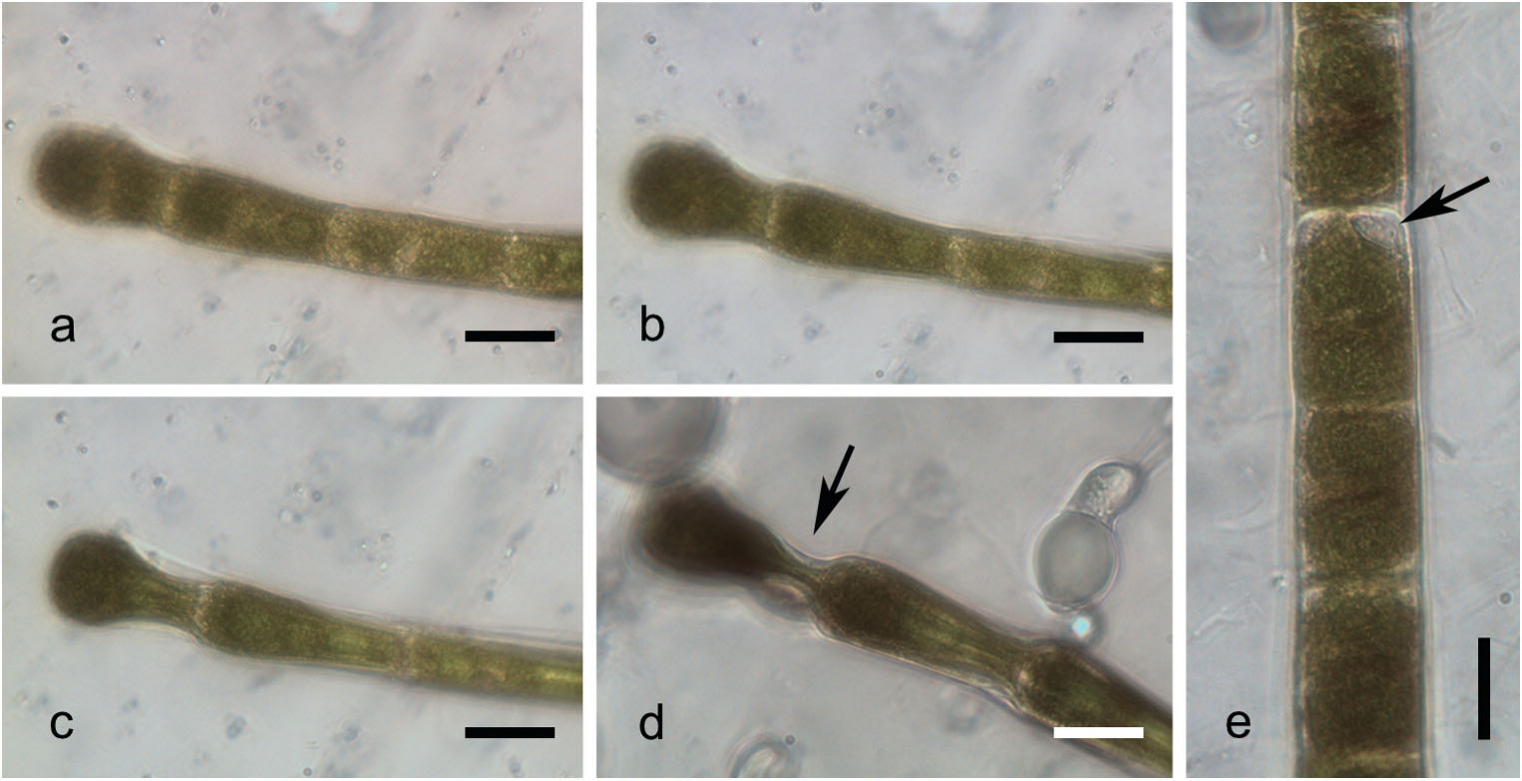
Microphotographs of young vegetative filaments of *Zygnema* sp. (strain MP2011Skan) taken during the process of freezing (10 min at – 10 °C) by a light microscope equipped with a cooled holder. **a**–**d** filament in the process of freezing showing the clavate shape of the cell (arrow); **e** thawed filament, showing a damaged cell with broken plasma membrane (arrow). Scale bar, 20 μm

Morphological changes to the cells were observed by cryo-microscopy during the freezing process (Fig. 5). The shape of the cells gradually changed from normal to clavate, especially in the terminal cell of the filament (Fig. 5a–d). All the cells in the filament were thicker at the same apical part and parallel bends appeared on squeezed cell walls (Fig. 5d; arrow). Cells recovered to their initial shape in a few seconds during thawing. When ice around the filament became softer, their cell walls returned to their original shape immediately. However, the protoplast formed a sphere in the middle of the cell and gained its normal shape only later on. Damage to the cells was visible after thawing; the protoplast was detached from the cell wall in several places (Fig. 5e; arrow) and the structure of the chloroplast was changed (Fig. 5e).

### Changes of the ultrastructure

**Fig. 6:**
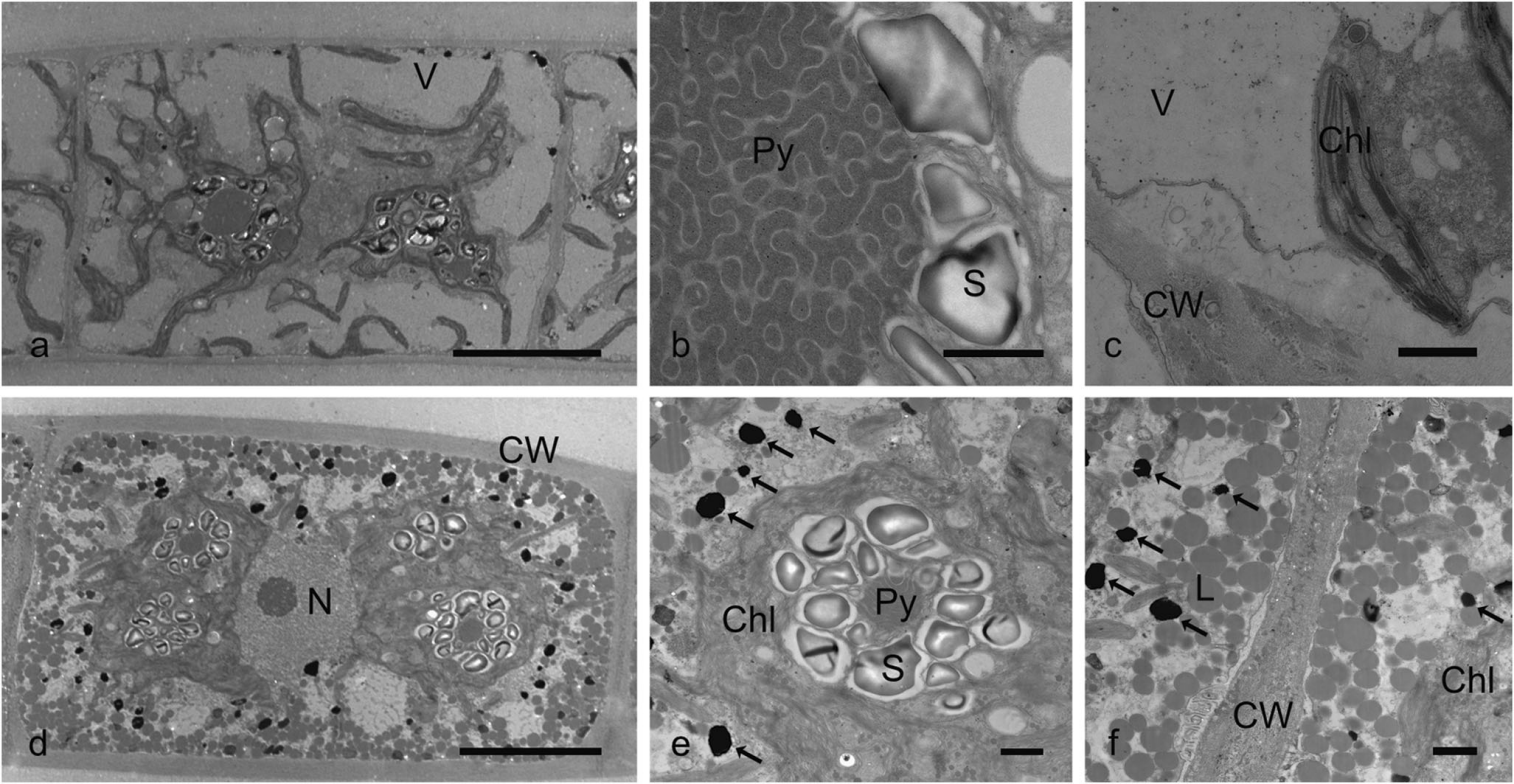
Transmission electron micrographs of *Zygnema* sp. (strain MP2011Skan) control cells. **a**–**c** Young vegetative cell (**a**, whole cell with vacuoles; **b**, pyrenoid with starch grains; **c**, chloroplast, cell wall, and vacuole). **d**–**f** pre-akinetes (**d**, whole cell with nucleus); **e**, chloroplast with reduced pyrenoid, electron-dense bodies (arrows); **f**, lipid bodies, thickened cell walls, electron-dense bodies (arrows). *Chl* chloroplast, *CW* cell wall, *L* lipid body, *N* nucleus, *Py* pyrenoid, *S* starch, *V* vacuole. Scale bars, 10 μm (**a**, **d**); 1 μm (**b**, **c**, **e**, **f**)

Young vegetative cells of *Zygnema* sp. showed a high degree of vacuolization (Fig. 6a). The two stellate chloroplasts contained pyrenoids with starch grains and were surrounded by a thin layer of cytoplasm (Fig. 6a). The center of large pyrenoids had a typical multilayer gyroid cubic membrane organization of the thylakoid membranes (Fig. 6b). The lamellar structure of thylakoid membranes was observed in chloroplasts (Fig. 6c). By contrast, pre-akinetes had thicker cell walls and little vacuolization (Fig. 6d). The size of the pyrenoids was reduced in comparison to young vegetative cells, and they also lacked the multilayer gyroid cubic membrane organization of the thylakoids. The thylakoid membranes in the small chloroplasts of pre-akinetes had a lamellar structure (Fig. 6e), whereas the cell periphery was full of lipid and electron-dense bodies (Fig. 6f).

**Fig. 7:**
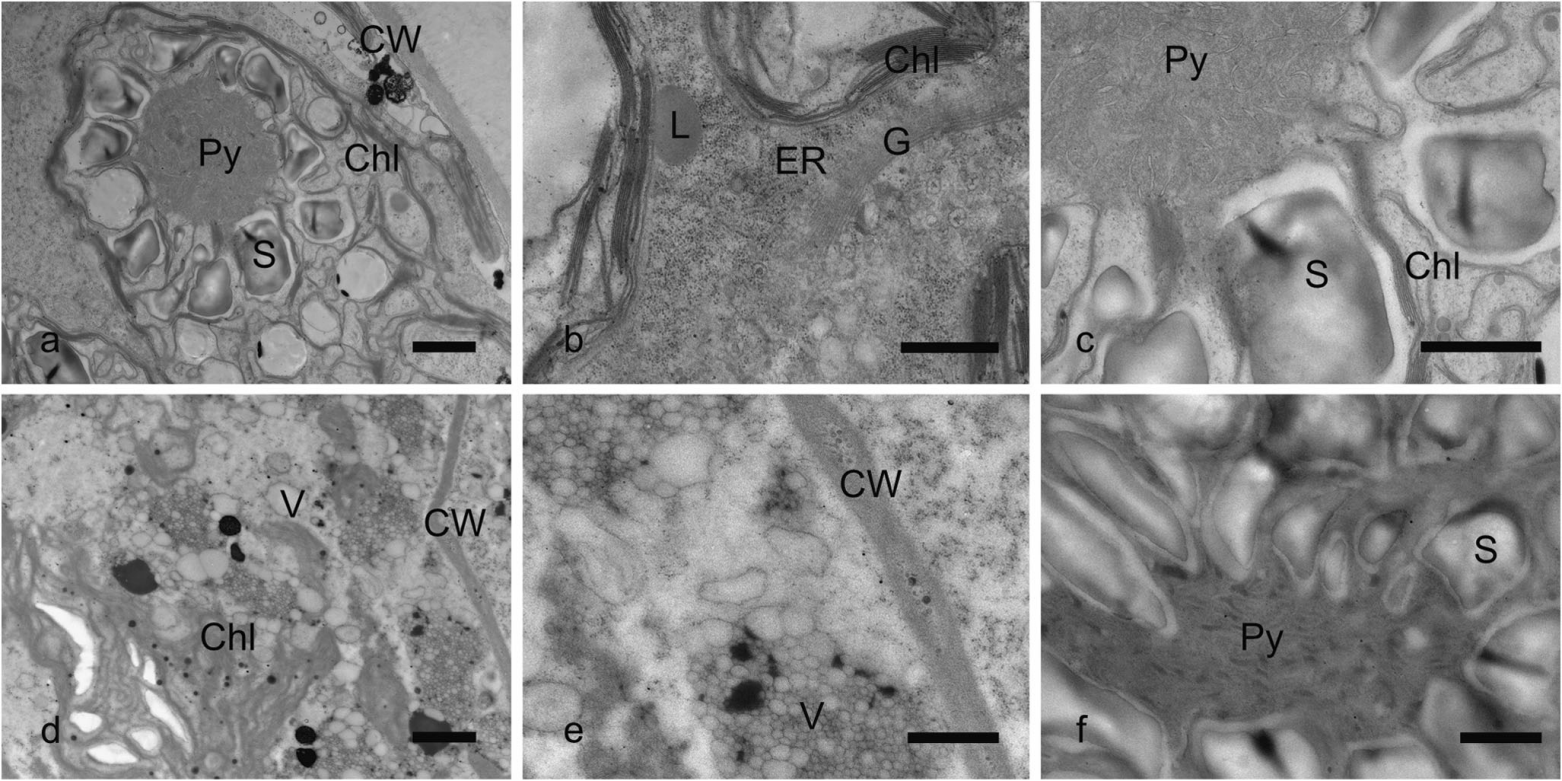
Details of transmission electron micrographs of *Zygnema* sp. (strain MP2011Skan) young vegetative cells after freezing to different temperatures. **a**–**c**, – 2 °C (**a**, chloroplast with pyrenoid and starch grains; **b**, detail of thylakoids and Golgi apparatus; **c**, detail of the pyrenoid). **d**–**f**, − 10 °C (**d**, cell with destroyed protoplast; **e**, detail of the foamy structure in the cell; **f**, detail of the pyrenoid). *Chl* chloroplast, *CW* cell wall, *ER* endoplasmic reticulum, *G* Golgi body, *L* lipid body, *Py* pyrenoid, *S* starch, *V* vacuole. Scale bars, 20 μm (**a**, **d**); 1 μm (**b**, **c**, **e**, **f**)

Young vegetative *Zygnema* sp. cells exposed to – 2 °C displayed minor modifications of the ultrastructure (Fig. 7a–c) when compared to control cells. Golgi bodies and endoplasmic reticulum remained intact (Fig. 7b). There were minor changes in the structure of the pyrenoid, organization of thylakoid membranes was altered, and no multilayer cubic membrane organization was observed (Fig. 7c). In contrast, exposure to – 10 °C (Fig. 7d–f) led to drastic ultrastructural changes in young vegetative cells (Fig. 7d–f). Protoplasts were full of small vacuoles, and the structure looked “foamy” particularly in the electron-dense compartments (Fig. 7d–e). The structure of the pyrenoid was damaged, and there was no sign of multilayer cubic membrane organization. The electron-dense thylakoid membranes in the pyrenoids displayed a lamellar structure (Fig. 7f).

**Fig. 8:**
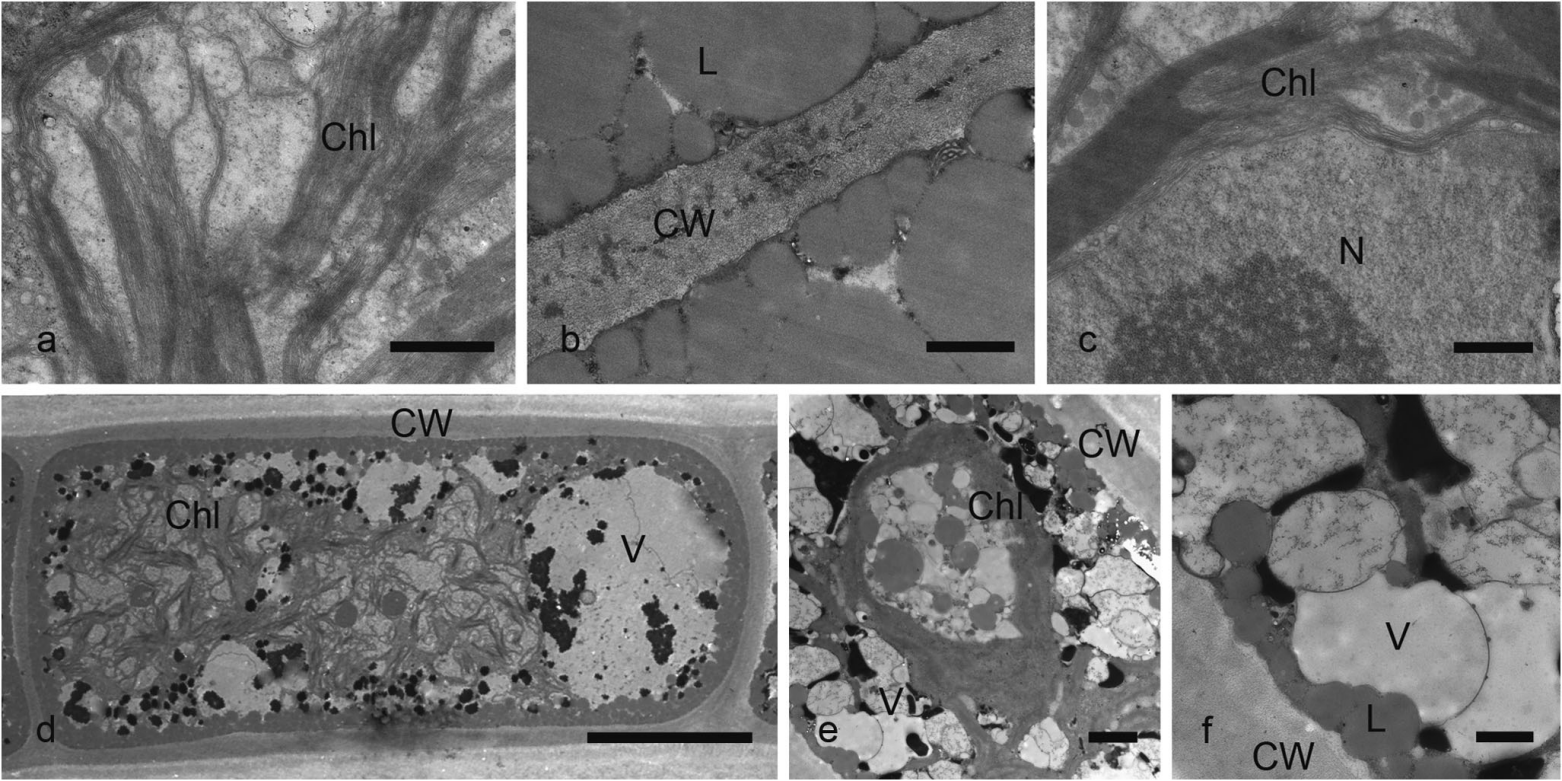
Details of transmission electron micrographs of *Zygnema* sp. (strain MP2011Skan) pre-akinetes after freezing to different temperatures. **a**–**c**, − 20 °C (**a**, detail of chloroplast and unchanged thylakoids; **b**, large lipid globules and thick cell walls; **c**, nucleus). **d**–**f**, − 70 °C (**d**, cell with damaged protoplast, large vacuole on the right side, and electron-dense bodies marked by arrows; **e**, chloroplast with destroyed structure and electron-dense bodies marked by arrows; **f**, detail of vacuoles in the protoplast, lipid bodies, and electron-dense bodies marked by arrows). *Chl* chloroplast, *CW* cell wall, *L* lipid body, *N* nucleus, *V* vacuole. Scale bars, 20 μm (**d**); 1 μm (**a**, **b**, **c**, **e**, **f**)

Pre-akinetes exposed to – 20 °C showed only minor changes in ultrastructure (Fig. 8a–c) when compared to control cells. The only observed difference was in the size of lipid bodies, most of which were drastically bigger than in controls (Fig. 8b). The structure of chloroplasts and the nucleus was the same as in untreated cells (Fig. 8a, Fig. 6d–f). As in the case of young cells, exposure to lower freezing temperatures had a more profound effect on pre-akinetes’ ultrastructure and their morphology changed substantially when exposed to – 70 °C (Fig. 8d–f). Overall, protoplasts contained large and multiple vacuoles (Fig. 8d), chloroplast ultrastructure was severely altered, the cytoplasm was full of vacuoles and electron-dense material (Fig. 8e), and lipid bodies accumulated at the periphery of the cell (Fig. 8f).

## Discussion

In this work, we investigated the limits of survival to frost by *Zygnema* sp. from the High Arctic region. We compared the freezing resistance of young vegetative cells and pre-akinetes. The latter are not specialized cells, but they gradually develop from young vegetative cells that stop dividing (Herburger et al. 2015). We report a substantial difference in the ability to deal with freezing temperatures between these two developmental stages. Young cells died at about – 6 to – 10 °C, whereas a small proportion of pre-akinetes could survive even down to – 70 °C. This corroborates our hypothesis because pre-akinetes are much more tolerant to desiccation than young vegetative cells (Pichrtová et al. 2014b).

### Limits to survival of young vegetative cells and pre-akinetes

Young vegetative cells were able to resist freezing temperatures, but frost drastically changed the ability to recover their photosynthetic activity. Except for cells at 0 °C and controls, all other cell samples failed to restore initial *Φ*_PSII_ values (Fig. 1). Thus, temperatures even slightly below zero (– 2 °C) negatively affected the cells’ physiology. At – 8 °C, the cells were unable to recover from severe frost damage as no *Φ*_PSII_ could be detected after 24 h of recovery. Hawes (1990) investigated freezing tolerance of *Zygnema* sp. cultures in exponential phase (i.e., young vegetative cells). They retained 92% viability after freezing to – 15 °C for 60 s, also showing a certain level of frost tolerance even in young cells. Comparable, the LT_50_ value of vegetative cells of *Tribonema bombycinum* (C. Agardh) Derbès & Solier after 5-day incubation was – 3 °C (Nagao et al. 1999). In addition, Nagao et al. (2008) found that 15% of young *Klebsormidium* cells survived freezing at – 10 °C.

In contrary to our findings, 12 tested strains of *Klebsormidium* survived experimental freezing to – 40 °C retaining 80% viability (Elster et al. 2008). However, the age of cultures used in the experiment was not given in the study. Similarly, all tested strains of Antarctic wetland green algae of the genera *Chlorella*, *Chlorosarcina*, *Pseudococcomyxa,* and *Klebsormidium* survived all freezing treatments (− 4 °C, − 40 °C, − 100 °C, and – 196 °C), even though the viability was reduced in comparison to control (on average, 56% at – 40 °C); (Šabacká and Elster 2006).

It is difficult to estimate the exact temperature limiting survival. Here, cell viability was estimated by two different methods; indeed, this is the first study that uses staining and measures of chlorophyll fluorescence of PSII together for this purpose. The LT_50_ value calculated based on the number of young viable cells (− 8.6 °C) was lower than the one calculated based on *Φ*_PSII_ (− 5.9 °C). However, these two values are not comparable. The first LT_50_ value indicated the temperature at which 50% of cells did not show any metabolic activity. The second LT_50_ represented the freezing temperature whereby chlorophyll fluorescence of the living cells was at 50% of the maximum 24 h after the experiment. It should be noted that staining was performed immediately after the end of the experiment to capture the cells’ status right after thawing. The decline in *Φ*_PSII_ observed after 24 h of recovery indicated that the cells’ photosynthetic machinery was severely damaged and, indeed, cells died soon afterward. Frost has many damaging effects, including changes to the ultrastructure of cell membranes, loss or fusion of membrane bilayers, and organelle disruption (Mazur 2004). Thylakoids are profoundly affected by frost (Garber and Steponkus 1976) and the cells’ survival depends on their ability to recover from the damage. Therefore, we believe that the LT_50_ value determined from *Φ*_PSII_ reflects the physiological status of the cells more realistically than auramine O staining.

In contrast to young vegetative cells, pre-akinetes showed astonishingly high freezing resistance. The LT_50_ value determined from a number of viable cells was − 26.1 °C and we counted 28.6% of cells to be alive after freezing to – 70 °C. Whereas none of the frozen samples was able to fully recover *Φ*_PSII_, a few viable cells were nevertheless found in a culture previously exposed to – 70 °C. This indicates that a small proportion of pre-akinetes survives even when its fluorescence cannot be detected. The present findings confirm the results of Hawes (1990), who tested long-term exposure of *Zygnema* sp. to – 20 °C. The small proportion of cells they found alive after long-term exposure to frost were most likely resistant pre-akinetes. Similar freezing resistance was found with akinetes of *Tribonema bombycinum* (C. Agardh) Derbès & Solier. Cells incubated under nutrient-limiting conditions formed resistant akinetes capable of surviving below – 30 °C (Nagao et al. 1999). Interestingly, both *Tribonema* (Xanthophyceae) and *Zygnema* sp. share the same life strategy even though they are phylogenetically unrelated; *Zygnema* sp. belongs to Archeplastida and *Tribonema* sp. to Sar (Burki et al. 2016; Brown et al. 2018).

We also investigated the effect of repeated freezing on young cells. Elster and Komárek (2003) showed that freezing down to – 4 °C was common during the polar summer periods and even at that temperature, the liquid content of the cells was not frozen so that species could survive without frost injuries. As shown by Hawes (1990), field-collected *Zygnema* sp. maintained its photosynthetic capacity without any cryoinjury during repeated overnight exposures to – 4 °C. In our experiments, repeated freezing to – 4 °C and – 6 °C had only a minor effect on *Φ*_PSII_ values. However, *Φ*_PSII_ dropped markedly after 24 h of recovery, as in the case of single freezing. In addition, exposure to – 8 °C resulted in 71.5% viable cells in the single freezing experiment but only 16.4% in the double freezing experiment. Thus, the results clearly show that repeated freezing can harm young vegetative cells more than a single freezing event. In the case of this experiment, we had low statistical support regarding the effect of temperature on *Φ*_PSII_, possibly due to fewer tested levels of this factor.

### Frost injuries

The most prominent differences between young vegetative cells and pre-akinetes included the size of vacuoles, chloroplast shape, and the massive occurrence of lipid bodies in pre-akinetes (Pickett-Heaps 1975; Pichrtová et al. 2014b; Pichrtová et al. 2016a). Frost injuries incurred during the experiments represented classical examples of freezing cytorrhysis, with deformed cell walls and retracted protoplasts (Pearce 2001; Beck et al. 2007; Buchner and Neuner 2010). They were similar to the plasmolyzed pre-akinete cells observed by Pichrtová et al. (2014a). However, damages derived from strong mechanical stress during freezing experiments were more severe than those observed in only plasmolyzed cells. As shown by cryo-microscopy, cells lost water during freezing cytorrhysis, but the cell walls remained flexible. Cells were squeezed completely during continuous freezing to – 10 °C. However, unlike protoplasts, cell walls remained unharmed and cells restored their shape.

Morris and McGrath (1981) observed the formation of gas bubbles during fast thawing of *Spirogyra grevilleana* (Hassall) Kützing filaments. The solubility of gasses increases during cooling and is predicted to double between 0 and – 20 °C (Hobbs 2010). Accordingly, when liquid water is removed to form ice, gasses remain concentrated in the residual solution. Small gas bubbles may become trapped between ice crystals when intracellular ice is formed. During thawing, they fuse before returning onto solution in the protoplast. Authors have not observed any remaining gas bubbles during slow warming (less than 0.5 K min^-1^). However, they applied much more moderate freezing temperatures than those used in the present study with pre-akinetes.

The ultrastructure of young cells and pre-akinetes of *Zygnema* sp. corroborated earlier studies (e.g., Kaplan et al. 2013; Pichrtová et al. 2013). The effect of freezing in young cells was clearly visible, as judged by the appearance of small foamy vacuoles. Freezing injuries affected mainly vacuoles and the structures of chloroplasts in young cells, whereas whole protoplast content seemed to be harmed by mechanical damage (Fig. 7d–e). Electron-dense bodies were present in young cells and particularly in pre-akinetes (Fig. 8d). They tended to fuse in pre-akinetes after exposure to – 70 °C and had an irregular shape (Fig. 8d–f). Lipid bodies, too, showed the same agglutination behavior; however, this could be an effect of their physical properties rather than a stress response. Liquid water is removed from the cells during freezing, which means that other compounds get closer to each other and may merge.

### Mechanisms of freezing protection

Lipids accumulate during the formation of pre-akinetes (Herburger et al. 2015; Pichrtová et al. 2016a). Accumulation of storage compounds represents a strategy for dealing with environmental stresses for many algae (Morison and Sheath 1985; Meindl et al. 1989; Karsten and Holzinger 2012; Herburger et al. 2016). They serve as a source of energy and carbon when optimal conditions are restored.

Freezing resistance is also triggered by the accumulation of organic osmolytes which are produced to prevent water loss and maintain homeostasis during osmotic stress. *Klebsormidium flaccidum* (Kützing) P.C.Silva, K.R.Mattox & W.H.Blackwell increases the content of sugars and other compounds during cold acclimation (Nagao et al. 2008). *Zygnema* collected from the Antarctic contained sucrose, with traces of glucose, fructose, and mannitol (Hawes 1990). Recently, production of organic osmolytes (sucrose) in *Zygnema* sp. upon desiccation stress has been analyzed by a transcriptomic approach, and several crucial pathways have been found (Rippin et al. 2017).

Other protective mechanisms involve safeguarding the cell wall from mechanical damage. Callose is located in the corners of cell walls, where the biomechanical forces are greatest during desiccation; a similar mechanism could also be involved in frost protection (Herburger and Holzinger 2015). In addition, mucilage layers abundant in pre-akinetes (Herburger et al. 2015) reduce water loss from cells and play a role in desiccation and freezing resistance (Knowles and Castenholz 2008).

In addition, prior work has documented the importance of hardening for stress resistance by *Zygnema* pre-akinetes. Naturally slowly desiccated pre-akinetes are resistant to severe osmotic stress (Pichrtová et al. 2014a) and pre-akinetes grown under mild desiccation stress are resistant to very fast desiccation (Pichrtová et al. 2014b). In contrast, our results showed that pre-akinetes induced by nutrient starvation were highly resistant to freezing. Indeed, additional frost hardening might even strengthen their resistance. Similarly, in another streptophytic alga, *Klebsormidium flaccidum* (Kützing) P.C.Silva, K.R.Mattox & W.H.Blackwell, survival rates after freezing increased by 70% after exposure to 2 °C for 7 days (Nagao et al. 2008).

### Dynamics of *Zygnema* mats and influence of frost in early spring

The current findings confirmed the hypothesized increased freezing resistance of pre-akinetes. However, the experiment was set to investigate rather short-term freezing (10 h), whereas polar winters last many months. Longer exposure to frost was previously found to lead to a gradual decrease in viability of *Zygnema* sp. cells at – 3 °C, whereas viability was lost rapidly at – 20 °C (Hawes 1990). Frozen pre-akinetes are metabolically active right after thawing and they start to divide immediately (Pichrtová et al. 2016b), transforming back to young vegetative cells and losing their freezing resistance. In addition, repeated freezing turned out to be more harmful to cells than single freezing. If frost or freeze-thaw cycles occur unpredictably during spring, young vegetative cells are severely damaged or die, which is probably the main cause of the perennial character of the mats (Pichrtová et al. 2016b).

On Svalbard, ground surface temperatures on a study site covered by snow dropped to – 30 °C several times during winter and the temperature oscillated around zero during spring and autumn indicating the occurrence of freeze-thaw cycles (Láska et al. 2012). Nevertheless, the accumulation of snow and ice offers very effective thermal insulation (Hawes 1989). While the air temperature of the Antarctic study sites fell to – 25 °C, the temperature under thick ice (30–50 cm) was only – 4 °C (Hawes 1989). Young vegetative cells could potentially survive the winter underneath snow and ice. So far, though, they have not been observed during the winter season.

## Conclusion

The production of pre-akinetes is a crucial strategy for overwintering in *Zygnema* sp. In accordance with our hypothesis, pre-akinetes were able to resist extreme temperatures substantially below the freezing point, whereas young vegetative cells turned out to be more susceptible to unexpected frost events. Development of young vegetative cells in the beginning of the spring season is crucial for rapid growth during the short summer season and the future of a population. Nevertheless, young dividing cells can be easily harmed by frost, which could endanger the whole population. Further research, e.g., long-term freezing or winter field observations are required to fully understand stress resistance by this alga.

## Electronic supplementary material

Online resource 1 Settings of the three experimental freezing cycles. (PDF 146 kb)
