# Supplementary material for: The conjugating green alga Zygnema sp. (Zygnematophyceae) from the Arctic shows high frost tolerance in mature cells (pre-akinetes)
Source: Protoplasma. 2019 Jul 10;256(6):1681–94. doi: 10.1007/s00709-019-01404-z (PMC6820810; doi:10.1007/s00709-019-01404-z)
Supplement: Supplementary file 1 — Settings of the three experimental freezing cycles. (PDF 146 kb) [file 709_2019_1404_MOESM1_ESM.pdf]

The conjugating green alga *Zygnema* sp. (Zygnematophyceae) from the Arctic shows high frost tolerance in mature cells (pre-akinetes)

Kateřina Trumhová[1], Andreas Holzinger[2], Sabrina Obwegeser[2], Gilbert Neuner[2], Martina Pichrtová[1*]

[1]Charles University, Faculty of Science, Department of Botany, Benátská 2, 128 00 Prague, Czech Republic

[2]University of Innsbruck, Institute of Botany, Functional Plant Biology, Sternwartestraße 15, 6020 Innsbruck, Austria

[*]Corresponding author: Martina Pichrtová, martina.pichrtova@natur.cuni.cz

	Young cells – single freezing	Young cells – double freezing	Pre-akinetes
Start temperature	1 °C	1 °C	1 °C
Settling time	45 min	45 min	45 min
Cooling rate	4 °C/h	4 °C/h	4 °C/h
Target temperature	0 °C, -2 °C, -4 °C, -6 °C, -8 °C, -10 °C, -12 °C, -14 °C	-4 °C, -6 °C, -8 °C	-10 °C, -20 °C, -30 °C, -40 °C, -50 °C, -60 °C, -70 °C
Exposure time	10 h	8 h	8 h
Warming rate	4 K/h	4 K/h	4 K/h
Final temparature	5 °C	5 °C	5 °C
Number of cycle repetitions	one	two	one

Online resource 1
